# How to accelerate the inorganic materials synthesis: from computational guidelines to data-driven method?

**DOI:** 10.1093/nsr/nwaf081

**Published:** 2025-03-04

**Authors:** Yilei Wu, Xiaoyan Li, Rong Guo, Ruiqi Xu, Ming-Gang Ju, Jinlan Wang

**Affiliations:** Key Laboratory of Quantum Materials and Devices of Ministry of Education, School of Physics, Southeast University, Nanjing 211189, China; Key Laboratory of Quantum Materials and Devices of Ministry of Education, School of Physics, Southeast University, Nanjing 211189, China; Key Laboratory of Quantum Materials and Devices of Ministry of Education, School of Physics, Southeast University, Nanjing 211189, China; Suzhou Laboratory, Suzhou 215004, China; Key Laboratory of Quantum Materials and Devices of Ministry of Education, School of Physics, Southeast University, Nanjing 211189, China; Key Laboratory of Quantum Materials and Devices of Ministry of Education, School of Physics, Southeast University, Nanjing 211189, China; Key Laboratory of Quantum Materials and Devices of Ministry of Education, School of Physics, Southeast University, Nanjing 211189, China; Suzhou Laboratory, Suzhou 215004, China

**Keywords:** inorganic materials synthesis, machine learning, computational guidelines

## Abstract

The development of novel functional materials has attracted widespread attention to meet the constantly growing demand for addressing the major global challenges facing humanity, among which experimental synthesis emerges as one of the crucial challenges. Understanding the synthesis processes and predicting the outcomes of synthesis experiments are essential for increasing the success rate of experiments. With the advancements in computational power and the emergence of machine learning (ML) techniques, computational guidelines and data-driven methods have significantly contributed to accelerating and optimizing material synthesis. Herein, a review of the latest progress on the computation-guided and ML-assisted inorganic material synthesis is presented. First, common synthesis methods for inorganic materials are introduced, followed by a discussion of physical models based on thermodynamics and kinetics, which are relevant to the synthesis feasibility of inorganic materials. Second, data acquisition, commonly utilized material descriptors, and ML techniques in ML-assisted inorganic material synthesis are discussed. Third, applications of ML techniques in inorganic material synthesis are presented, which are classified according to different material data sources. Finally, we highlight the crucial challenges and promising opportunities for ML-assisted inorganic materials synthesis. This review aims to provide critical scientific guidance for future advancements in ML-assisted inorganic materials synthesis.

## Teaser

State-of-the-art machine learning techniques and computational methods are transforming the synthesis of inorganic materials, providing innovative solutions to accelerate advances of experimental synthesis.

## INTRODUCTION

With the rapid development of various multiscale simulation methods such as density functional theory (DFT), an in-depth understanding of the relationship between the microstructure and macroscopic properties of materials has been established from a theoretical perspective. To date, computational materials science has emerged as an important and growing area within materials science [1–3]. Theoretical calculations have the potential to rapidly identify materials with outstanding performance from the vast and unexplored chemical space. However, due to the difficulty and complexity of chemical reactions, the synthesis of newly predicted materials may require months of repeated experiments. In addition, a substantial number of theoretically predicted materials with promising properties have often proved to be difficult or even impossible to synthesize. It is therefore crucial to identify materials with high synthesis feasibility and to determine the optimal experimental conditions for synthesis in the laboratory [4].

Unlike organic synthesis, the mechanisms involved in inorganic solid-state synthesis processes remain unclear [5]. Since the universal theory on phase evolution during heating is lacking, and synthesis processes contain a multitude of adjustable parameters (such as temperature, reaction time, and precursors), most chemists rely on chemical intuition to identify the optimal experimental conditions. During the process of identifying new functional materials, chemists often review scientific literature related to the synthesis of similar materials and repurpose existing formulations. The identified similar materials and synthesis methods are typically constrained by chemical intuition and idiosyncratic human decision-making, which impedes the design of synthesis experiments for unexplored materials. Moreover, the limited experimental resources of typical laboratories and hard-to-control variables present significant challenges to exploring all potential combinations of experimental conditions. Furthermore, some materials with promising properties are frequently thermodynamically metastable, which renders the synthesis through conventional solid-state synthesis approaches challenging [6]. Consequently, the typical material discovery cycle, which relies on a trial-and-error approach, often takes months or even years.

Due to the lack of applicable principles and universal physical models for evaluating the synthesis feasibility of inorganic materials, the charge-balancing criterion is frequently employed as one of the empirical evaluation conditions [7,8]. This criterion has the capacity to remove materials that do not exhibit a net neutral ionic charge under common oxidation states of the constituent elements. Although this criterion is derived from ‘prior’ physicochemical knowledge, it is unable to accurately predict inorganic materials with high synthesis feasibility. Among experimentally observed Cs binary compounds listed in the Inorganic Crystal Structure Database (ICSD), only 37% meet the charge-balancing criterion under common oxidation states [9]. The charge-balancing criterion does not consider the diverse bonding environments between atoms in different classes of materials, such as ionic materials, metallic alloys, and covalent materials. This deficiency leads to suboptimal predictions of the synthesis feasibility of inorganic materials. Another commonly employed methodology involves applying DFT calculations to obtain the formation energy of the crystal structure compared to the most stable phase within the chemical space [10]. The underlying assumption of this method is that synthesizable materials do not have any decomposition products with thermodynamic stability. However, due to the neglect of kinetic stabilization and barrier, it is challenging to predict the synthesis feasibility of materials based solely on the formation energy [4,11]. Heuristic models derived from thermodynamic data such as reaction energies have the potential to predict favorable reactions and pathways for material synthesis [12–14]. To date, methods utilized to understand and predict materials synthesis encompass *in situ* X-ray diffraction (XRD) [15], *ab initio* molecular dynamics simulations [16], and optimization of experimental conditions via machine learning (ML) techniques [17,18].

As a data-driven technique, ML can bypass time-consuming first-principles calculations and experimental synthesis and uncover the process/structure-property relationships, which has emerged as a powerful tool in the field of material science. Recently, ML techniques have been successfully utilized for the automatic design and reverse synthesis of organic molecules, leveraging the retention and transformation of functional groups [19,20]. For instance, Gao *et al.* developed a neural network model for recommending experimental conditions for organic reactions, demonstrating good performance on common examples across different reaction classes [21]. Maser *et al.* developed relational graph convolutional networks to predict experimental conditions for substrate-specific cross-coupling reactions [22]. ML techniques exhibit good capability to identify compounds with high synthesis feasibility and to recommend suitable experimental conditions for chemical reactions. Nevertheless, in contrast to ML-assisted organic synthesis [23], the utilization of ML techniques in inorganic materials synthesis remains a nascent field of research. The primary obstacles to be overcome are the scarcity of data and the class imbalance issue, which are caused by the complexity and high cost of experimental synthesis. These factors significantly impede the development of ML-assisted inorganic material synthesis.

**Figure 1.**
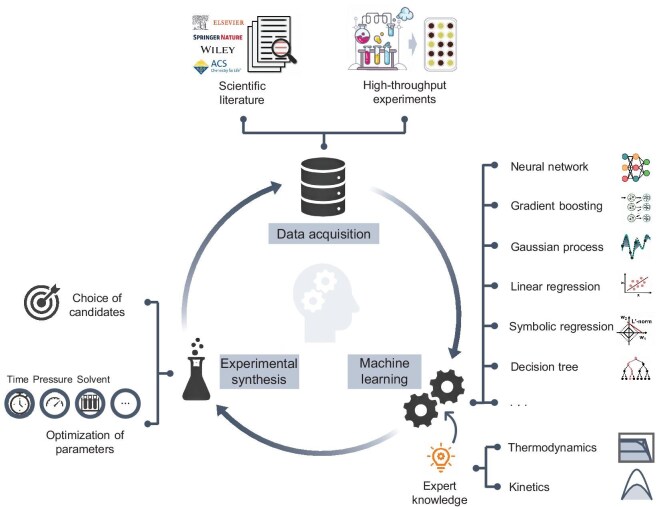
Schematic illustration of computation-guided and machine learning (ML)-assisted inorganic materials synthesis. The diagram illustrates the interplay among data acquisition, machine learning, and experiment synthesis. Physical models such as thermodynamics and kinetics can be integrated into ML models as expert knowledge, effectively improving model performance and interpretability.

In this review, we focus on computation-guided and ML-assisted inorganic materials synthesis, as well as discuss recent progress for the acceleration and optimization of experimental synthesis (Fig. 1). We begin with the introduction of common synthetic strategies for inorganic materials. Following that, we introduce physical models based on thermodynamics and kinetics, as well as computational applications relevant to the synthesis feasibility of inorganic materials. Subsequently, we discuss data acquisition, commonly utilized material descriptors and ML techniques in ML-assisted material synthesis. Then we review ML applications for accelerating and optimizing inorganic material synthesis. Finally, we conclude with crucial challenges and prospective research directions to facilitate the application of ML techniques in inorganic material synthesis.

## COMMON SYNTHESIS METHODS FOR INORGANIC MATERIALS

**Figure 2.**
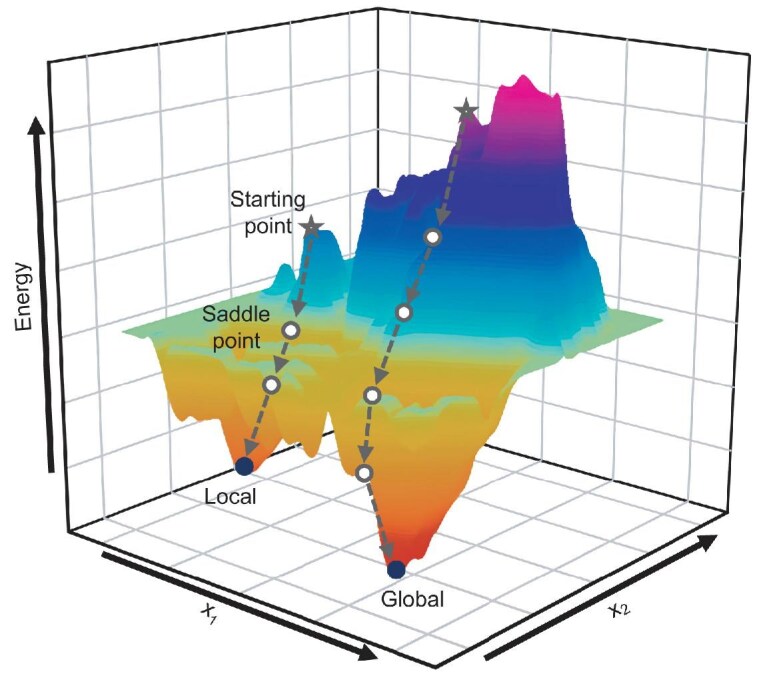
Free energy surface with a global minimum alongside numerous local minima, which describes the relationship between the energy of the system and various system parameters x_i_ (such as atomic configurations, temperature, and pressure). Gray stars, gray dots, and dark blue dots represent starting points, saddle points, and energy basins in different reaction pathways, respectively.

From the thermodynamic perspective, the synthesis process of materials can be regarded as the formation of a target metastable or stable material from a mixture of precursor materials with thermodynamically stable phases [24]. The energy landscape of materials provides insight into the relationship between the energy of different atomic configurations and various parameters such as temperature, demonstrating the stability of possible compounds and their reaction trajectories (Fig. 2). From different starting points, the free energy of the system can decrease along with different reaction pathways, ultimately falling into different free energy basins. When the system moves from one minimum to another, energy barriers need to be overcome. Reaction energies involved in the solid-state synthesis of inorganic materials, including nucleation energies and activation energies for diffusion, are directly related to the energy barriers in the materials landscape. Based on the classical nucleation theory, the formation of crystals mainly involves two steps, namely, nucleation and growth [25]. Nucleation is the initial step in crystal formation, where atoms and molecules self-assemble into a new thermodynamically stable phase. Although the bulk phase is thermodynamically more stable with respect to the initial phase, the increased energy of the interface between the initial phase and the new phase leads to an activation energy. Following nucleation, crystal growth depends on the rate of diffusion and the chemical reactions occurring at the surface and interface. The diffusion process, which requires overcoming activation energies, enables atoms to move from one stable bonding environment to another due to concentration gradients during the growth of crystals.

The synthesis of inorganic materials is a complex process involving the interaction of numerous atoms, structures, and phases, resulting in the absence of universal synthesis principles. Consequently, a variety of synthetic methods have been developed to overcome reaction energies and move on to the desired free energy minimum in the energy landscape. In this review, we selectively overviewed three main synthetic methods for inorganic materials.

### Direct solid-state reaction

One of the most prevalent methodologies for synthesizing inorganic materials is the direct reaction of solid reactants [26]. Direct reactions frequently occur at elevated temperatures, involving contact reaction, nucleation, and crystal growth at the interface between solids. The initial stage of reactions involves contact between particles of the reactants, which is followed by a chemical reaction on the surface. This results in the formation of a new phase, accompanied by structural adjustment and crystal growth of products. For certain metal compounds, the initiation of the reaction in the melt or the rapid condensation of vapor phase necessitates the application of extreme conditions, such as high temperature and pressure. This method does not require the utilization of solvents, which are easy to handle and can be produced on a large scale, yielding highly crystalline materials with few defects and high stability. However, only the most thermodynamically stable phases can crystallize under the high temperature and long heating time [27]. The synthesis protocol of this method typically requires several days of heating and repeated grinding to achieve a uniform mixture of reagents. The control of particle size in the synthesized material is challenging, resulting in microcrystalline structures with irregular sizes and shapes [12,28]. To get insight into the solid-state reaction process, *in situ* powder X-ray diffraction has been employed in order to detect the different intermediates and products [29].

### Synthesis in the fluid phase

One of the challenges associated with direct solid-state reactions is the difficulty in ensuring uniform mixing of reagents, which can lead to a reduction in reaction rates and an increase in reaction times. An effective and common solution is using fluid phase to facilitate the diffusion of atoms and increase the reaction rates [30]. The convection effects and active stirring within the fluid reaction medium facilitate the diffusion of atoms. During the nucleation process of synthesis in the fluid phase, the nucleus with the smallest activation energy is observed to form first, despite the potential for it to lack the most stable thermodynamic phase. Thus, the rate-limiting step in synthesis in a fluid phase is nucleation. Moreover, phase evolution in fluid reactions is a significant factor in the transition process from reactants to products. At the initial stage, kinetically stable compounds are formed rapidly, followed by the nucleation of some more stable compounds. During the growth of the nucleus, a reduction in the concentration of specific materials in solution results in the dissolution of previously formed compounds. Therefore, it is crucial to understand and investigate factors influencing the nucleation process and required reactants, such as the relative solubility and structures of potential products. The fluid phase includes various types, such as a solvent, a melt, a eutectic flux, a mineralizer, and a reactive flux. The available reaction mediums encompass a range of options, including water solutions, organic or non-aqueous solvents, sols and gels, salts with low melting points, and so on. The hydrothermal method is one of the representative synthesis methods in a fluid phase, which utilizes water solutions as the reaction medium in the tailored closed reaction vessel [27]. Chemical reactions occur at elevated temperature and pressure, after which post-treatment techniques are employed, including separation, washing, and drying. The solvothermal method has been developed based on the hydrothermal method, differing in the utilization of organic or non-aqueous solvents instead of water. The application of elevated temperatures and pressures may facilitate reactions that are challenging to achieve under typical conditions, leading to the formation of unique material structures. Numerous attempts have been made for low-dimensional metal materials synthesis by hydrothermal/solvothermal methods [31–33]. Advantages of this approach include simplicity of operations, low cost, and capability of synthesizing nanocrystals with unique morphologies and excellent properties. In addition, other representative methods, such as the sol-gel method [34,35] and the molten salt method [36], also demonstrate superior capabilities in synthesizing inorganic materials.

### Synthesis by chemical vapor deposition

Chemical vapor deposition (CVD) is a process whereby solid materials are deposited from vapors through chemical reactions that occur on or near the surface of a heated substrate [37,38]. The formation of thin films in CVD is primarily driven by gas-phase and gas-solid chemical reactions [39]. CVD holds the potential to precisely control the morphology of films, achieve rapid material deposition rates, and ensure high deposition purity. Due to thermodynamic and kinetic constraints and the limitations in the flow of gaseous reactants and products, the CVD process is often complex [40]. In addition, films are usually deposited under high temperatures, limiting the substrates that can be partially coated. Moreover, high temperatures might induce stresses in films deposited on materials with varying coefficients of thermal expansion, leading to mechanical instability of the deposited films.

## COMPUTATION-GUIDED INORGANIC MATERIAL SYNTHESIS

In the realm of material synthesis, a pivotal and complex field, the synthesis feasibility of materials is directly aligned with the results observed in synthesis experiments. Outcomes obtained from experimental synthesis, which reflect the success or failure of synthesis experiments, can be directly treated as the target properties for ML models. However, due to the high resource demand and time cost, collecting material synthesis data by performing large-scale synthesis experiments is both economically and practically challenging. While the stability of materials obtained by DFT calculations cannot be used to directly determine the synthesis feasibility, stability at 0 K has a significant impact on the outcomes of synthesis experiments. Furthermore, the physicochemical descriptors derived from thermodynamic and kinetic models with DFT calculations enable the assessment of materials synthesis feasibility, which can provide guidance for inorganic material synthesis to a certain extent.

### Phase diagram and descriptor for inorganic material synthesis

**Figure 3.**
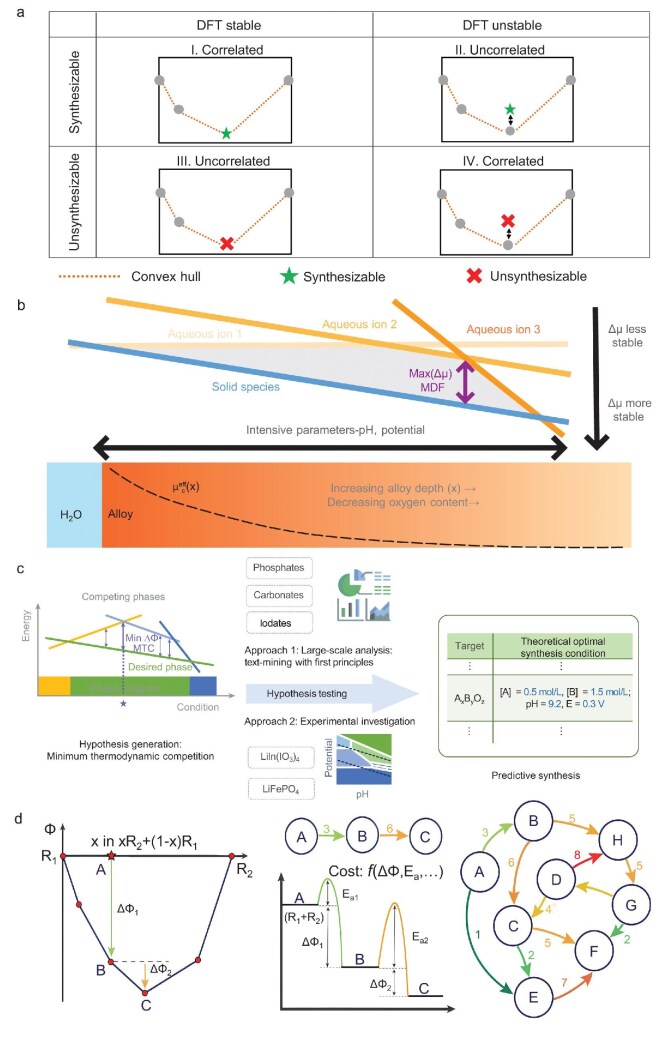
(a) Material classification matrix related to the synthesis feasibility and thermodynamic stability of materials. Reproduced with permission from ref. [44]. (b) Descriptors based on thermodynamic driving forces to quantitatively evaluate the formation of solid phase in aqueous environments. Reproduced with permission from ref. [48]. (c) Schematic diagram for the platform to predict the conditions of aqueous solution-based synthesis. Reproduced with permission from ref. [49]. (d) Three models of chemical reactions in the thermodynamic phase space, in which the degree of abstraction of chemical reactions increases from left to right. Reproduced with permission from ref. [13].

The stability of materials can be generally assessed by performing DFT calculations, which is related to their synthesis feasibility. The formation energy and energy above the convex hull (*E*_hull_) are common assessments of the thermodynamic stability of materials [41]. The formation energy is defined as the energy released during the synthesis of a material from its constituent elementary substances. The formation energy of a material is inversely proportional to its stability, with negative formation energies characteristic of stable materials. The stability of materials at 0 K is typically evaluated using DFT calculations, which serve as a crucial initial step in assessing the feasibility of experimental synthesis. The convex hull of a given material can be constructed by computing the free energies of different components. *E*_hull_ represents the energy difference between the energy of the material and convex hull. Materials located on the convex hull line are considered stable, whereas those exhibiting *E*_hull_ values within the range of 0 meV/atom to 100 meV/atom are classified as metastable. As rough assessments of synthesis feasibility, the formation energies and *E*_hull_ of materials are extensively calculated and stored in open-access databases such as Material Project (MP) [10] and in scientific literature. For instance, Emery *et al.* have provided an exhaustive database of 5329 cubic and distorted perovskites, which contains formation energy and *E*_hull_ by performing DFT calculations [42]. Bartel *et al.* have utilized data available in the MP to construct the convex hull, thereby obtaining the decomposition enthalpy of 85 014 inorganic crystalline solids [43]. However, it should be noted that not all computationally stable materials can be synthesized, not all computationally unstable materials cannot be synthesized. Lee *et al.* created a material classification matrix to explore the relationship between the synthesis feasibility and thermodynamic stability of materials (Fig. 3a), demonstrating that the synthesis feasibility of materials cannot be precisely predicted via their thermodynamic stability obtained from DFT calculations [44].

The phase diagram of materials is closely correlated with their synthesis feasibility. The phase diagram provides information regarding the most stable phase as various factors change, including temperature, pressure, and chemical composition, which can effectively regulate the pathways of chemical reactions [15]. Based on the computed phase diagram, Miura *et al.* synthesized MgCr_2_S_4_ thiospinel via a thermodynamically favorable pathway of exchange reactions [45]. Bianchini *et al.* predicted the first formed phase in the synthesis of P2-type Na_0.67_MO_2_ (M=Co, Mn) by minimizing the unconstrained reaction energy. The results suggest that the formation of the initial phase might have a significant influence on the dynamics of subsequent reactions and the final phase selectivity [46]. Nevertheless, predicting, characterizing, and mapping free energies of metastable materials pose significant challenges. The initial crucial step is to identify the global and local minima of the energy landscape in the configuration space using efficient structure optimization algorithms, which correspond to the ground state and metastable states of materials, respectively. Subsequently, mapping free energy surfaces of metastable phases as functions of various thermodynamic state variables (e.g. pressure, temperature, and composition) becomes a crucial step. However, for a considerable number of metastable configurations, the associated computational costs become prohibitively high. It is therefore necessary to develop alternative models for the calculation of free energies that are both cost-effective and accurate. Once the equation of states for all phases have been calculated, the final task is to classify and identify phase interfaces and regions of metastable equilibrium. The thermodynamic parameters obtained by DFT calculations can be used to develop the hyperdimensional chemical potential space, which provides the minimum thermodynamic competition among intermediates and potential reactions. For instance, Todd *et al.* calculated the chemical potential space for Y-Mn-O systems and attributed the formation of Y_2_Mn_2_O_7_ to the chemical potential distance of Na-based intermediates. The map of calculated chemical potential in the thermodynamic space of materials can assist researchers in comprehending selective reactions in the vast and multi-dimensional compositional spaces [47].

The application of descriptors derived from the thermodynamic model enables the measurement of material synthesis. Large and negative differences between chemical potentials can lead to huge thermodynamic driving forces. This phenomenon is responsible for the formation and evolution of the initial phase, as well as the establishment of the final equilibrium microstructure. Walters *et al.* employed thermodynamic driving forces as the descriptor to conduct a quantitative evaluation of the formation of a solid phase in aqueous environments (Fig. 3b). This descriptor is developed using the free energies of solids and ions, which can effectively measure the difficulty of forming solid oxides in metal-water systems [48]. Wang *et al.* proposed a minimum thermal competition (MTC) strategy to optimize pathways of solution-based material synthesis (Fig. 3c) [49]. The experimental procedures of solution synthesis are collected from extensive scientific literature, and the thermodynamic optimal points predicted by the MTC physical model have a strong correlation with experimental synthesis conditions.

### Thermodynamic model with kinetic features

The synthesis of novel functional inorganic materials often stems from the interplay between thermodynamics and kinetics. The global or local minimum in the energy landscape, which depends on the boundary conditions of a particular system, represents the thermodynamic equilibrium or kinetically stable state of the system. Fig. 3d illustrates a purely thermodynamic model, a thermodynamic model by incorporating simple kinetic behavior, and a chemical reaction network obtained by incorporating further thermodynamic/kinetic features, respectively. This demonstrates the increasing degree of abstraction of chemical reactions from left to right [50]. Furthermore, the dynamical simulations of materials also have the potential to predict their synthesis feasibility. The phase space of materials can be sampled by solving the classical Newton's equations of atomic motion. This approach is useful for investigating the evolution of materials in the phase space and obtaining different properties of materials at non-zero temperature based on statistical methods. Dynamical simulations are valuable tools for gaining insights into the formation process of crystalline materials from the kinetic perspective. The forces of interatomic interaction can be obtained through a variety of methods, including DFT calculations and empirical force fields. In recent decades, significant advancements related to the mechanisms of bounded solid reactions at the atomic level have been made, including molecular dynamics (MD) [51] and a dynamical Monte Carlo-based method known as Kinetic Monte Carlo (KMC) [52]. Reaction force fields can be employed to investigate the mechanisms and dynamical parameters of specific chemical reactions, which can further break chemical bonds [53,54]. In addition, models based on the KMC method can approximate the rates of chemical reactions by performing quantum mechanical calculations, thereby holding the potential to explore partial regions of atomic potential energy surfaces. For instance, Kim *et al.* utilized atomistic simulations to investigate the growth process of dolomite CaMg(CO_3_)_2_, and revealed that dolomite initially precipitates a cation-disordered surface, where high surface strains inhibit further crystal growth [55]. Zeng *et al.* utilized the reaction energy to predict and control polymorph selectivity in solid-state reactions, which influences the role of surface energy in promoting the nucleation of metastable phases [28]. Szymanski *et al.* proposed a quantitative framework based on the max-Gibbs energy to predict the initial product formed during solid-state reactions [56]. Despite these efforts, the simulation of the intricate dynamic processes involved in the synthesis of inorganic materials remains a significant challenge, largely due to the extended timeframes and vast spatial scales inherent to solid periodic systems.

## ML MODELING IN INORGANIC MATERIAL SYNTHESIS

### Data acquisition

**Figure 4.**
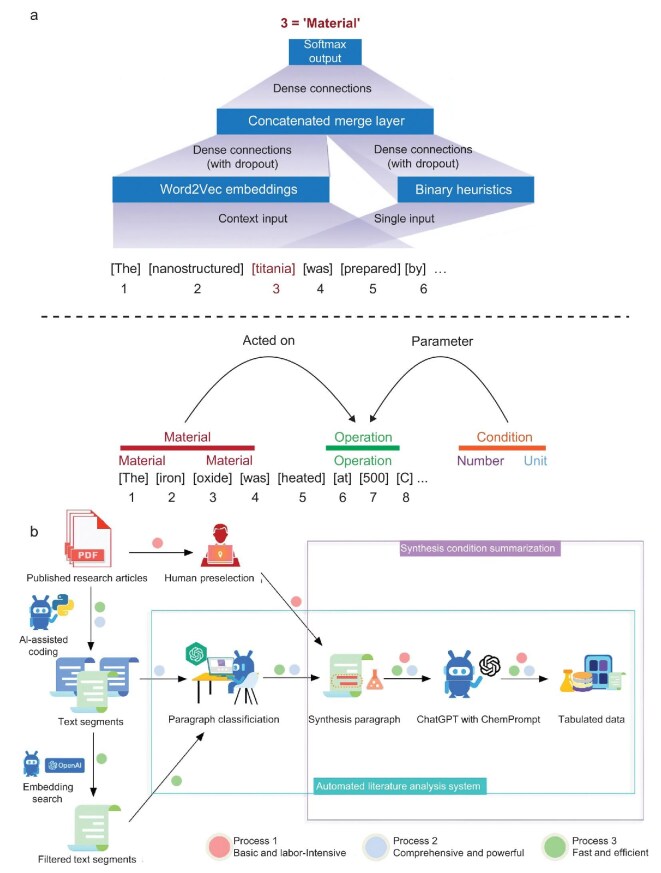
(a) Neural-network and parse-based synthesis parameter extraction. The hierarchical neural network assigns word labels (e.g. ‘MATERIAL’) by processing word embedding and heuristic vectors. The grammatical parse of sentences is utilized to resolve word-level labels (below colored bar) into sequential word-chunk–level labels (above colored bars). Reproduced with permission from ref. [65]. (b) Schematics of the ChatGPT Chemistry Assistant workflow employing ChatGPT and ChemPrompt for efficient text mining and summarization of MOF synthesis conditions. Reproduced with permission from ref. [67].

The quality and quantity of datasets are the cornerstone of ML projects, directly influencing the predictive accuracy and generalization capacity of ML models. The collection of a high-quality and comprehensive dataset is a prerequisite for the training of reliable ML models. For material synthesis, the dataset for ML models is primarily sourced from published scientific literature and high-throughput experiments. A significant quantity of material data with diverse characteristics is stored in scientific literature, serving as a potential data source for ML. To date, millions of papers related to inorganic materials synthesis have been published, which can provide useful information for the optimization of synthesis procedures and the prediction of experimental conditions. Due to the great amount of scientific literature related to material synthesis, manually extracting information from literature is an enormous and time-consuming task. Developing various data mining tools and algorithms to collect and process textual information to replace manual extraction, enables rapid extraction of required information from an extensive number of articles. The application of automated data extraction techniques has the potential to facilitate the rapid search of scientific literature and the efficient construction of a comprehensive material database [57]. This can provide an extensive space for large-scale data mining and analysis. For instance, IBM employed text mining techniques to construct a database with more than 10 000 recipes from Bon Appétit [58]. Despite their different domains of application, the process of preparing food can be considered analogous to materials synthesis. For substantial textual and graphical information related to experimental synthesis in scientific literature, automated data extraction techniques can automatically download articles, then extract key information related to material synthesis. Subsequently, the extracted information is encoded into a synthesis dataset and aggregated according to material systems. This approach has been demonstrated to be effective in the rapid conversion of human-readable articles into machine-readable synthesis parameters and synthesis planning routes. To date, numerous attempts have been carried out to automatically collect and process textual information from extensive articles related to experimental synthesis [59–63,64]. For instance, Kim *et al.* collected more than 640 000 articles related to material synthesis and trained ML models with natural language processing algorithms to analyze these articles (Fig. 4a) [65]. Yang *et al.* annotated 305 articles related to polycrystalline material synthesis and constructed a dataset. The corpus was obtained through a two-step manual labeling process, which ensures high quality and the inclusion of novel scientific information in material science [66].

Additionally, the information available in scientific literature is frequently presented in various formats, which poses a challenge in collecting and preprocessing diverse textual and graphic information from published scientific literature. The heterogeneity of data is reflected not only in different forms (e.g. tables, text, and figures) but also in the diversity of data structures, measurement units, and material systems. Therefore, data extraction techniques should convert these discrete and non-standardized data elements into the dataset with coherent and uniform formats. Moreover, due to the complexity of synthesis processes, the unclear physicochemical mechanisms and the ambiguity of natural language might hinder the collection of material synthesis datasets. In addition to handling different formats, data extraction techniques should have the potential to categorize material synthesis data into three levels, i.e. synthetic methodologies (the highest level), experimental procedures (intermediate level), and synthesis parameters (the lowest level). In practice, rule-based algorithms can be developed by analyzing grammar and keywords in sentences, which have the capability to distinguish three levels of material synthesis [68]. Furthermore, information-dense word embeddings have proven effective in capturing underlying physicochemical knowledge and structure-property relationships in materials science [69]. In addition, a range of techniques have emerged as powerful tools for extracting structural information including ChemDataExtractor [70] and ChemTagger [71], which employ a combination of heuristic–rule-based approaches and natural language processing techniques. With the rise of large language models, this technique has revolutionized the natural language processing fields. Large language models have the potential to answer domain-specific questions and search information from scientific literature and databases, showcasing significant promise for application in materials science [72]. Zaki *et al.* utilized a GPT-based framework that bridged the gap between scientific literature and the domain-specific synthesis database, achieving high accuracy in the parsing and processing of articles [73]. Zheng *et al.* developed a workflow that incorporated various text mining techniques based on ChatGPT to extract experimental parameters for metal-organic framework synthesis from scientific literature (Fig. 4b) [67].

Another common approach for data acquisition in material synthesis is employed by high-throughput experiments. This approach employs automation and parallel processing techniques to rapidly synthesize and screen materials. The parallel execution of multiple experiments enables multiple samples to be synthesized simultaneously, accelerating the synthesis and screening process of new materials. A substantial number of samples can be explored in a relatively short period, which significantly increases experimental efficiency and is crucial for obtaining a large-scale dataset. Moreover, the utilization of automated equipment and robots in high-throughput experiments can automatically handle various experimental steps including reaction stirring, automated liquid handling, and temperature control, thus improving the efficiency of material synthesis [74]. Automated experiments are also conducive to reducing experimental errors as well as increasing the precision and consistency of experiments, which aids in obtaining reliable data and mitigates the impact of manual operations. However, due to the high cost of conducting experiments, problems of data scarcity often exist. Although datasets from high-throughput experiments encompass both negative and positive material data, the hard-to-control variables and idiosyncratic human selection may result in subjective preferences. These subjective preferences exhibit not only in the data distribution but also on the material data that researchers can obtain.

### Representation

The performance of ML models also heavily relies on the feature set. Material systems can be converted into numerical representations, which are defined as features or descriptors. Appropriate material features allow for developing the relationship between structure/processing and synthesizability. Good feature sets should provide appropriate description of material characteristics and be closely related to the target properties, with the potential to facilitate the development of clear relationships between structure/processing and synthesizability. In general, effective material features should meet the following criteria: (1) features should uniquely characterize materials and fundamental processes linked to target properties; (2) materials that exhibit significant differences should be described by features that demonstrate comparable magnitudes of difference; (3) features should avoid reliance on time-consuming first-principles calculations and experiments, enhancing convenience and ease of use; (4) the dimensions of feature sets should be minimized while preserving the accuracy of ML models.

To date, a substantial number of material features have been developed. Among these features, one widely utilized category is composition-based features, which can provide elemental information about compounds, including atomic number, atomic radii, and Mendeleev number. Given that composition-based features are well-suited for material systems with constrained structural degrees of freedom, such as perovskites, it is essential to incorporate features with additional information into the feature sets of ML models for complex tasks. The composition of each structure may comprise a variety of configurations, necessitating a detailed description of the crystal structure. The features used to describe the crystal structure should remain invariant under translations, rotations, and permutations of atoms. The most commonly utilized structural features include the Coulomb matrix, atomic cluster expansion (ACE), smooth overlap of atomic positions (SOAP), and crystal field interaction descriptor (CFID) [75]. In addition, graph-based features have been developed to describe the connection of atoms in molecules and crystal, such as distance matrix and crystal graph convolutional neural networks (CGCNN) [76]. The structure of molecules and crystals can be represented as a graph containing vertices and edges, where vertices and edges represent atoms and chemical bonds, respectively.

The synthesis process of materials contains multiple variables such as reaction temperature, precursor concentration, and pressure, significantly influencing the outcomes of experiments. Thus, for ML models aimed at material synthesis, features pertaining to experimental parameters are also of paramount importance. Typical experimental parameters, such as precursor solution concentration, temperature, and pressure, can be directly integrated into ML models. For an ML classifier to classify the CVD-grown MoS_2_ samples belonging to the categories of ‘can grow’ and ‘cannot grow’, features related to the synthesis process (e.g. gas flow rate) and reactions (e.g. reaction temperature) were collected as the initial feature set [77]. The trained ML model can predict the synthesis feasibility of MoS_2_ samples based on a given set of CVD parameters and recommend optimal conditions. Furthermore, data-driven techniques, such as symbolic regression, can also be employed to effectively develop descriptors related to target properties [78]. For instance, based on the sure independence screening and sparsifying operator (SISSO) algorithm, Bartel *et al.* identified an accurate and simple descriptor to predict Gibbs energy, which is a key factor in determining the stability of materials and the equilibrium conditions of reactions [79].

### ML algorithm selection

In general, ML can be classified into three main categories: supervised learning, unsupervised learning, and reinforcement learning. In practice, the selection of ML algorithms is contingent upon the specific requirements of the task at hand. Supervised learning employs labeled data to train ML models with common algorithms including logistic regression [80], linear regression [81], support vector machine [82], etc. Unsupervised learning employs ML techniques to identify intrinsic patterns within unlabeled datasets, involving algorithms such as principal component analysis [83] and clustering [84]. Both supervised and unsupervised learning evaluated the performance of ML models through the minimization of loss functions or objective functions. Moreover, semi-supervised learning exists between supervised and unsupervised learning, employing both labeled and unlabeled data [85]. Reinforcement learning differs significantly from both supervised and unsupervised learning, which deals with the interaction between an agent and its environment [86]. The objective of reinforcement learning is to maximize cumulative rewards, with well-known algorithms including variational methods, Monte Carlo learning, and Markov chains.

## APPLICATIONS OF ML-ASSISTED INORGANIC MATERIAL SYNTHESIS

### ML-assisted material synthesis from scientific literature

**Figure 5.**
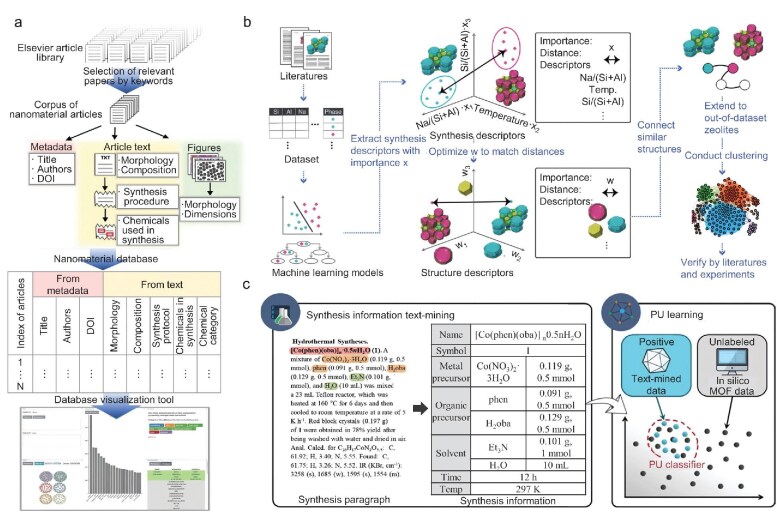
(a) Schematic diagram of the process of mining structural information from scientific literature for the synthesis of nanomaterials. Reproduced with permission from ref. [88]. (b) Schematic diagram of linking structure and synthesis descriptors in zeolites. ML models are developed based on experimental data, mapping the synthesizable domains onto the multidimensional phase diagram. Reproduced with permission from ref. [89]. (c) Strategy of mining scientific insights on metal-organic framework synthesis from articles. The proposed text-mining strategy can extract useful experimental parameters (highlight sections) from published papers. Reproduced with permission from ref. [90].

To date, extensive scientific literature related to material synthesis contains rich information and can serve as an important source for materials synthesis data. Chen *et al.* collected synthesis pathway data from both scientific literature and experiments, and developed an ML-based framework to assess the probability of synthesizing single-phase VO_2_ under given conditions prior to synthesis [87]. Lee *et al.* integrated calculated thermodynamic stability and composition-based descriptors to train ML models for predicting synthesis feasibility of ternary compounds with a half-Heusler structure [44]. Hiszpanski *et al.* developed scientific article-processing tools to extract information related to nanomaterial synthesis from 35k articles. Based on the collected synthesis protocols, the correlation between reagents and the nanomaterial morphologies and compositions was identified (Fig. 5a) [88]. Muraoka *et al.* collected synthesis records of zeolites from scientific literature and conducted an in-depth analysis to gain physicochemical insights using ML techniques that the correlation between the structural similarity and synthesis conditions of zeolites were evaluated by integrating graph theory and a clustering algorithm. Further crossover experiments indicated the synthesis similarity of zeolites and uncovered the synthesis-structure relationship (Fig. 5b) [89]. Park *et al.* employed rule-based algorithms to extract different experimental parameters (such as precursors, solvents, time, and temperatures) from 28 565 papers pertaining to metal-organic framework (MOF) synthesis. The developed text-mining code can convert published papers into a plain text format and identify paragraphs related to the experimental synthesis, then extract crucial information related to synthesis experiments such as chemicals (highlighted in Fig. 5c) from those paragraphs. By applying experimental conditions as input, trained ML models can successfully predict the synthesis feasibility of target MOF materials [90].

**Figure 6.**
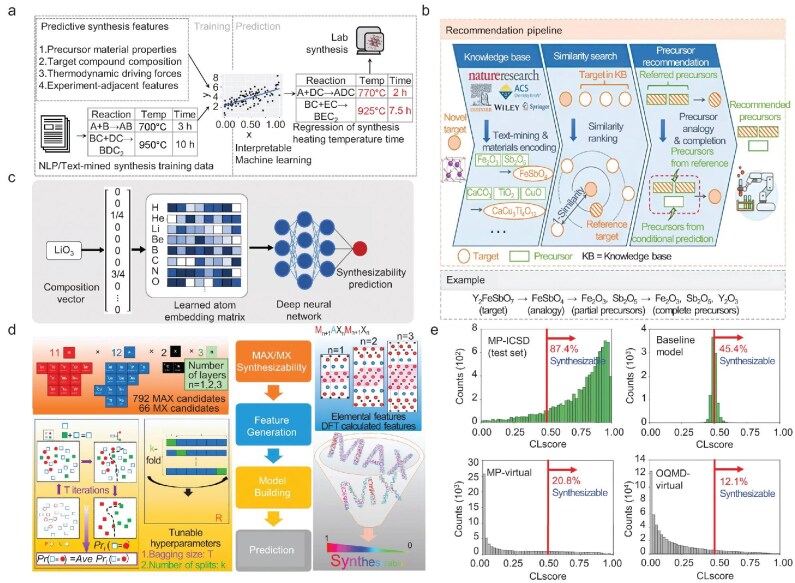
(a) Schematic illustration of prediction conditions of solid-state synthesis by applying ML techniques. Based on large-scale text-mined synthesis dataset, interpretable ML models revealed two crucial conditions for solid-state reactions: heating temperature and time. Reproduced with permission from ref. [91]. (b) Schematic diagram of precursor recommendation pipeline, which contains three steps: (1) identify target materials from scientific literature through text-mined techniques; (2) rank target materials according to the material similarity; (3) recommend precursors based on the similarity to the most similar target. Reproduced with permission from ref. [92]. (c) Model architecture of the deep learning-based model for material synthesizability. Reproduced with permission from ref. [9]. (d) Chemical search space diagram and computational work path including positive and unlabeled ML (PU learning), which predicts the theoretically proposed 2D materials with high synthesis feasibility. Reproduced with permission from ref. [93]. (e) The crystal-likeness scores predicted through PU learning for experimentally synthesized materials in Material Project database. Reproduced with permission from ref. [11].

In addition to synthesis of the specific material systems, ML models have been developed to expedite and optimize synthesis of materials based on large historical materials-science datasets. Huo *et al.* constructed a large-scale solid-state synthesis database from the scientific literature by employing information retrieval and natural language processing techniques. An inductive ML approach is utilized to mine the physicochemical knowledge hidden in articles and to accurately predict the experimental parameters of inorganic material synthesis (Fig. 6a) [91]. Kim *et al.* collected published synthesis recipes by utilizing natural language processing, then developed an automated platform to capture key insights into the crucial experimental parameters driving the synthesis of specific materials. In comparison to heuristic strategies, transfer learning exhibits a superior capacity to predict the outcomes of experimental synthesis [59]. Based on the chemical similarity of materials, He *et al.* integrated text-mined techniques and self-supervised learning to develop a recommendation strategy for the selection of a precursor in experimental synthesis. A knowledge base containing 29 900 solid-state synthesis recipes has been constructed through the application of text-mining techniques. This recommendation strategy is capable of effectively selecting precursors for the experimental synthesis of new inorganic materials (Fig. 6b) [92]. Antoniuk *et al.* developed a deep learning synthesizability model (SynthNN) to investigate the synthesis feasibility of inorganic materials in the entire chemical space. Compared to 20 expert material scientists, SynthNN exhibits superior performance in the head-to-head material discovery comparison (Fig. 6c) [9].

Furthermore, datasets pertaining to experimental synthesis, as gathered from scientific literature, frequently exhibit a class imbalance issue. In particular, these datasets often contain only materials that were successfully synthesized, with few samples of failed synthesis experiments. Information pertaining to unsuccessful synthesis experiments is frequently documented in unreported laboratory notebooks, which contain data essential for assessing the synthesis feasibility of materials. In this situation, semi-supervised learning represents an effective solution to the aforementioned problem. Semi-supervised learning is a ML technique that utilizes a small portion of labeled samples and lots of unlabeled samples to train ML models. The objective of this technique is using labeled samples to gain the function for predicting labels of unlabeled samples in the dataset. Positive and unlabeled (PU) learning is a semi-supervised ML algorithm that is widely utilized in materials science. The assumption of this algorithm is that each unlabeled sample could correspond to either the positive or negative category. For material synthesis, the labeled and unlabeled samples correspond to the synthesized materials and unknown materials, respectively. For instance, Frey *et al.* employed PU learning to predict 2D metal carbides and nitrides (MXene) materials with high synthesis feasibility (Fig. 6d) [93]. Subsequently, PU learning was utilized to classify the synthesis feasibility of materials in the MP database (Fig. 6e) [11].

### ML-assisted material synthesis from high-throughput experiments

**Figure 7.**
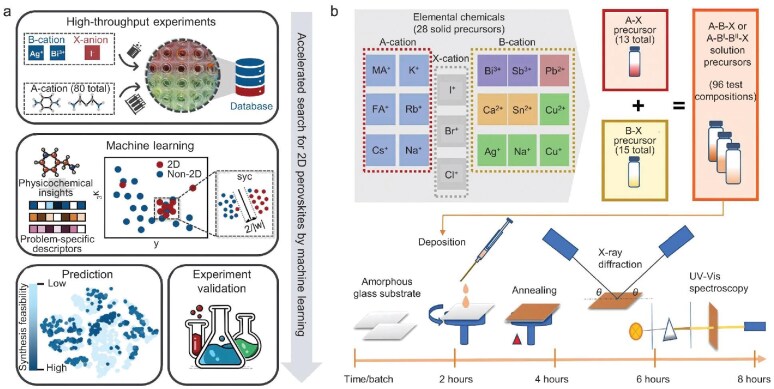
(a) Framework of ML-assisted two-dimensional silver/bismuth perovskite synthesis, which integrates high-throughput experiments, ML techniques, and physicochemical insights. Each step is represented by a gray box. Reproduced with permission from ref. [94]. (b) The optimized experimental workflow for synthesizing and characterizing perovskite-inspired target compositions including thin-film deposition, X-ray diffraction, and UV-visible spectroscopy measurements. Reproduced with permission from ref. [95].

The implementation of high-throughput experimentation within a laboratory setting allows for the generation of comprehensive datasets comprising sufficient material data, facilitating the discovery and property optimization of materials. High-throughput experiments can rapidly test numerous material compositions, experimental conditions, and processing methods of material synthesis, thereby holding the potential for large-scale screening of materials within a short period of time. ML models can elucidate the structure/process-synthesis relationship hidden in the material dataset generated by high-throughput experiments, thereby enabling the prediction of properties of new materials and the optimization of synthesis experiments. In practice, the selection of appropriate ML techniques and features, based on the specific synthesis methods, can assist in designing synthesis pathways and optimizing experimental parameters, thereby increasing the success rate of material synthesis. For instance, Wu *et al.* proposed a framework aimed at accelerating solution synthesis of two-dimensional silver/bismuth (2D AgBi) iodide perovskites, which integrates small-scale high-throughput experiments, previous physicochemical insights and ML techniques (Fig. 7a). Based on the interaction between inorganic and organic components in 2D hybrid organic-inorganic perovskites, a set of material features related to synthesis of 2D AgBi iodide perovskites are developed by quantifying physicochemical, stereochemical, and topological properties of organic precursors. Subgroup discovery techniques are utilized to identify the region more favorable for the formation of 2D perovskite structures, and an equation capable of quantitatively evaluating the synthesis feasibility of 2D AgBi perovskites is proposed by employing ML techniques. Finally, the success rate of the synthesis feasibility has been increased by a factor of four relative to traditional approaches, which provides a practical route for solving multidimensional chemical acceleration problems with a small dataset from a typical laboratory with limited experimental resources available [94].

Sun *et al.* employed a neural network to assist structural analysis for outcomes in experimental synthesis, accelerating the synthesis of perovskite-inspired materials (Fig. 7b). A fully connected deep neural network is utilized to classify the dimensions of compounds obtained from high-throughput experiments, achieving an accuracy of 90% and a fast analysis time [95]. Tang *et al.* utilized fusion models with combinatorial fusion analysis to control the data quality of high-throughput experiments, which achieve increased predictive ability for the synthesis of hybrid organic-inorganic halide perovskites. Fusion models based on the combinatorial fusion analysis can not only identify previously unrecognized effects in chemical reactions, but also serve as effective tools for quality control in high-throughput experiments [96]. Du *et al.* devised and established a high-throughput synthetic workstation (more than 1000 experimental recipes), which integrates ML techniques to enhance the comprehension of potential mechanisms for controlling cluster size and optimize the experimental conditions of material synthesis [97]. Dahl *et al.* developed a framework integrating ML techniques and a joint spectral-kinetic model to comprehensively understand and accurately predict the formation process of nanomaterials [98]. Karpovich *et al.* combined a conditional variational autoencoder (CVAE) with high-throughput experiments to classify inorganic reactions and predict conditions of specific solid-state reactions [99].

Chemical vapor deposition is a widely utilized synthesis method for large-area and high-quality monolayer materials. The multivariational problem of synthesis optimization encompasses a multitude of experimental parameters, including temperature, precursor, and substrate, which heavily influence the quality and properties of experimental outcomes. Park *et al.* employed a ML model based on the Gaussian process to systematically analyze the synthesis data of monolayer hBN, successfully providing the relationship between multigrowth parameters and determining the growth window of domain size [100]. To solve problems that involve thousands of tunable parameters and a huge search space, Rajak *et al.* developed a ML scheme based offline reinforcement learning with a deep generative model of chemical reactions to predict the optimum conditions for the rapid synthesis of 2D MoS_2_ monolayers using chemical vapor deposition [86]. Ji *et al.* integrated high-throughput experiments coupled with random forest regression models to determine the optimal growth conditions for high-quality single-wall carbon nanotubes [101].

**Figure 8.**
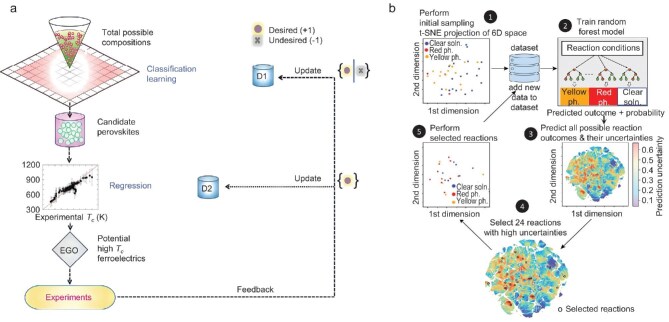
(a) A two-step strategy based on active learning techniques for rapid screening of PbTiO₃-based perovskites. One classification model is developed to screen perovskites with high synthesis feasibility, and another regression model is trained to predict the *T*_c_ for the candidate perovskites. Reproduced with permission from ref. [102]. (b) Framework of the diverse-mini-batch-sampling active learning loop for predicting the dimensionality of metal halide perovskites. Reproduced with permission from ref. [103].

Moreover, the synthesis of inorganic materials in typical laboratories requires extensive resources and a long time, consequently leading to a considerable financial outlay in the acquisition of synthesis data. Active learning can selectively label the most crucial and valuable samples, thereby enabling the effective improvement of ML model performance through the utilization of a limited number of labelled samples. Balachandran *et al.* employed a two-step strategy based on ML techniques to screen PbTiO₃-based perovskites with a high ferroelectric Curie temperature (Fig. 8a). A support vector classifier is trained to screen potential compositions of perovskites, followed by a support vector regressor combined with active learning to select perovskites for experimental synthesis and feedback. Results of both successful and failed experiments are utilized to iteratively modify ML models by active learning loops [102]. Li *et al.* introduced active learning into the high-throughput experimental process for material discovery, which aims at controlling and understanding phase formation in lead iodide perovskites with different dimensionalities (Fig. 8b) [103].

Furthermore, data from thermodynamic models can also be integrated into the ML framework, which can possibly improve the performance of ML models. Singstock *et al.* combined thermodynamic data obtained from first-principles calculations and experimental methods to investigate the synthesis feasibility of Chevrel phase compounds, in which newly generated material descriptors can well assess the thermodynamically stability by estimating decomposition enthalpy [104]. Sun *et al.* constructed a physically constrained sequential learning framework to identify stable organic-inorganic perovskite materials. Thermodynamic data from first-principles calculations is integrated with high-throughput degradation testing data, which is employed to train an end-to-end Bayesian optimization algorithm with probabilistic constraints [105]. Kaufmann *et al.* proposed an ML framework to evaluate the synthesis feasibility of disordered metal carbides, which is based on experimental and thermodynamic data [106]. Meng *et al.* combined high-throughput computations and experimental synthesis to propose descriptors for the formation of high-entropy alloys by applying ML techniques [107].

## AUTONOMOUS LABORATORY

**Figure 9.**
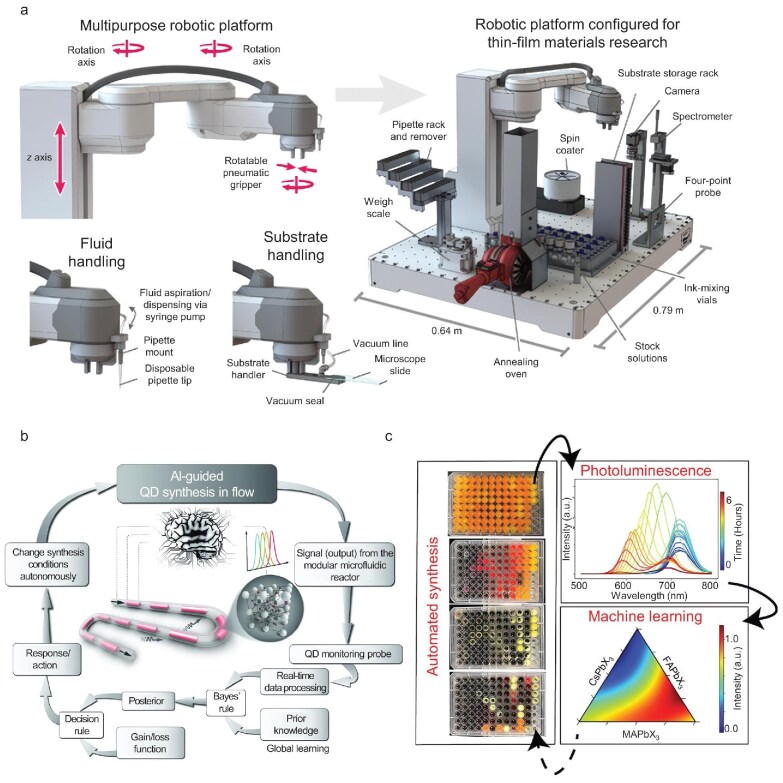
(a) Schematic illustration of the Ada self-driving laboratory based on the modular robotic platform. The right part indicates the experimental workflow for thin-film material research by the utilizing robotic platform. Reproduced with permission from ref. [110]. (b) Schematic illustration of the concept of AI-guided quantum dots synthesis employing modular microfluidic reactors. Reproduced with permission from ref. [114]. (c) Schematic of the automated workflow for the combinatorial discovery of perovskite materials. Reproduced with permission from ref. [116].

The autonomous laboratory represents a significant and emerging field within the discipline of materials science, offering a unique capacity to facilitate the discovery of novel functional materials [108,109]. In autonomous laboratories chemical experiments are automated, effectively increasing the efficiency of experiments. Moreover, ML techniques can automatically determine the optimal experimental parameters of a given chemical reaction based on previous experimental results, thereby guiding the flow of subsequent synthesis experiments. Currently, autonomous laboratories for energy material synthesis adhere to three principal paradigms. The first paradigm is the fully automated robot laboratory, where manual operations are replaced by fully automated robots, and the synthesis process is controlled by Bayesian optimization workflows (Fig. 9a) [110–112]. One example is the autonomous laboratory for the solid-state synthesis of inorganic powders, developed by Szymanski *et al*. This laboratory combined computations, historical data from scientific literature, ML techniques, and active learning to plan and interpret the outcomes of experiments performed using robotics [100]. The second paradigm is microfluidic systems, which explore the synthesis parameter space using modular microfluidic platforms based on integrated neural networks (Fig. 9b) [113–115]. The third paradigm combines manual operations with automated workflows to accelerate material preparation. For instance, Higgins *et al.* used a low-cost microfluidic pump robot to quickly prepare halide perovskite crystals via the antisolvent method, and adopted automated photoluminescence spectroscopy for characterizing the environmental stability of halide perovskite crystals and quantum dots (Fig. 9c) [116].

## CHALLENGES AND OUTLOOK

With the embrace of the era of big data and artificial intelligence (AI), the era of AI for science is positioned to emerge. AI for science is devoted to promoting powerful AI tools across every scientific and engineering domain, converting these techniques into the equal partners of modeling, simulation, and data analysis, which can aid researchers in formulating and addressing scientific problems. For material synthesis, the utilization of ML techniques is revolutionizing this domain, enabling the prediction of novel materials with high synthesis feasibility and optimization of experimental parameters, thereby accelerating the discovery of materials with desirable properties. Despite this promising outlook, the use of ML techniques to accelerate experimental synthesis remains a nascent and evolving field. Even the most state-of-the-art ML models are still unable to provide accurate predictions regarding optimal synthesis routes and experimental outcomes. To bridge the gap between ML/computation-guided strategy and actual synthesis experiments, both theorists and experimentalists are required to contribute their respective expertise and efforts towards this objective. From the theoretical perspective, using a ‘bottom-up’ strategy to construct mathematical models from the atomistic level for complex chemical synthesis processes can facilitate the deep understanding of thermodynamics and kinetics. In addition, physical quantities calculated from thermodynamic and kinetic models can be integrated into ML models as material features, which can improve the performance and interpretability of those models. From the experimental perspective, development of a high-quality experimental dataset is the prerequisite of seeking a global phenomenological description of similar processes (or their extended combinations), which can guide further atomic simulations and chemical synthesis. This review introduces computation-guided and ML-assisted inorganic material synthesis, as well as discussing well-established techniques and recent progress in this domain. Despite numerous attempts, ML-assisted inorganic material synthesis remains a relatively new and evolving area, with significant ambiguity and challenges. The following section will address some of the key issues and potential frontiers for further exploration.

### Data scarcity and imbalance

As data-driven techniques, the quantity and quality of the dataset is a fundamental prerequisite for the utilization of ML models in material science. As material synthesis data is obtained from experimental synthesis, a typical published work contains synthesis results of a few compounds. Due to the ongoing resource and time requirements of synthesis experiments, materials synthesis data from high-throughput experiments are also limited. Therefore, issues of data scarcity frequently occur during the preparation of the material synthesis dataset, hindering the use of ML algorithms. Consequently, it is of paramount importance to reduce the cost of labelling samples. Recently, the rapid development of active learning has brought promising prospects and innovative solutions to this dilemma [117]. Active learning can selectively obtain the most informative samples, demonstrating notable advantages in reducing the number of labeled samples and improving the quality of the dataset. Therefore, active learning is an economically beneficial approach that provides feasible solutions to accelerate inorganic material synthesis, particularly when sample labeling is expensive.

Another common issue encountered in the data acquisition of inorganic material synthesis is class imbalance. Two significant sources of material data for inorganic material synthesis are literatures and high-throughput experiments. Material synthesis data collected from literature exhibits strong bias towards materials that has been successfully synthesized (positive material data), while the vast majority of failed synthesis experiments (negative material data) are recorded in inaccessible laboratory notebooks. These unreported failure data contain critical information that is essential for determining the boundary between successful and failed synthesis experiments. For ML classification models to distinguish materials with high and low synthesis feasibility, both collected successful and failed experiments should be treated as the positive and negative class of the training set, respectively. Although high-throughput experiments provide both negative and positive material data, the data imbalance problem still exists due to the low success rate and subjective preferences from chemists, which is reflected in the data distribution and material data that we can obtain. It is therefore imperative that novel ML algorithms are developed to address the issue of unbalanced datasets in material science. In addition, materials synthesis data should be shared according to the FAIR (findable, accessible, interoperable, and reusable) principles, thereby helping researchers to build high-quality synthesis datasets.

### The ‘bottom-up’ strategy

Developing a bottom-up strategy for constructing mathematical models from the atomistic level for complex chemical synthesis processes requires a deep understanding of the underlying physicochemical principles. The synthesis of novel functional inorganic materials is frequently driven by the interaction between thermodynamic and kinetic processes. Material synthesis involves complex processes, which can be investigated by employing MD simulations. MD simulations can trace the movement of atoms in phase transitions, thereby providing atomistic-level details of synthesis processes. Classical MD simulation based on a force field is efficient, but the prediction error of its potential energy surface might be large. In contrast, *ab initio* MD simulation based on DFT calculations is accurate but limited to a few thousand atoms. Recently, ML simulations based on ML potentials can simultaneously achieve the high accuracy of *ab initio* MD simulation and the high efficiency of classical ML simulations. MD simulations based on ML potential have been utilized to investigate the nucleation and crystal growth processes. The objective of training ML potentials is to minimize the cost function in order to accurately describe the *ab initio* data. Among various ML potential models, those based on neural networks and gradient process regression potential are suitable for describing rare events including nucleation and growth, which have gained wide attention in the material modeling community [118]. To date, ML potentials have been successfully employed to investigate the phase transitions between crystalline, amorphous, and liquids. However, due to the complexity of the synthesis process, simulating the formation of inorganic materials with complex structures from precursors is still a very challenging problem.

For experimental chemists, determining the optimal synthesis route of new functional materials is a challenging task, with numerous potential avenues for failure. As a data-driven technique, ML might separate human from fundamental operators, such as the determination of experimental conditions, leading to the unreliable synthesis routes. Therefore, developing ML models with predictive ability and good interpretability can provide accurate prediction and reliable explanations at each step, which aids chemists to effectively identify potential risks. By integrating physical principles and domain knowledge with the powerful ML techniques, physics-inspired ML models offer enhanced interpretability and good predictive capability, providing reliable synthesis routes and in-depth understanding of mechanisms driving the synthesis process. In practice, the development of physics-inspired ML models requires the construction of appropriate material descriptors. Given the inherent complexity and time-consuming nature of inorganic materials synthesis, it is imperative to consider both the intrinsic properties of the given materials and experimental synthesis routes employed. On the one hand, the intrinsic properties of given materials and thermodynamic/kinetic features related to synthesis processes can be obtained by performing first-principles calculations. By incorporating these features derived from theoretical calculations, ML models hold the potential to provide accurate predictions and a reliable structure-synthesis feasibility relationship. On the other hand, experimental parameters related to material synthesis (i.e. temperature and pressure) often have a direct impact on the structure and properties of synthesized outcomes. Thus these parameters can be directly incorporated into ML models as features, facilitating the optimization of experimental synthesis routes and development of the process-synthesis feasibility relationship. Integrating theoretically-derived features and experimental parameters to develop physics-inspired ML models can achieve both good predictive ability and high interpretability for inorganic material synthesis, which is still ongoing.
